# Suicidal behaviour among patients with congestive cardiac failure in a Nigerian teaching hospital

**DOI:** 10.4102/sajpsychiatry.v30i0.2204

**Published:** 2024-04-23

**Authors:** Tomilola O. Shofu-Akanji, Bolanle A. Ola, Dapo A. Adegbaju, Adeola O. Ajibare, Adefemi A. Adeoye, Ismail O. Adesina

**Affiliations:** 1 Department of Clinical Services, Federal Neuropsychiatric Hospital Yaba, Lagos, Nigeria; 2 Department of Psychiatry, Lagos State University Teaching Hospital, Lagos, Nigeria; 3 Department of Cardiology, Lagos State University Teaching Hospital, Lagos, Nigeria

**Keywords:** congestive cardiac failure, heart failure, suicidal behaviour, suicidal ideation, suicidal attempt, sociodemographic factors, clinical factors

## Abstract

**Background:**

Suicidal behaviour is an established psychiatric complication of congestive cardiac failure (CCF), contributing significantly to morbidity and death by suicide. The magnitude and risk factors for suicidal behaviour among patients with CCF are yet to be unpacked, especially in developing nations such as Nigeria.

**Aim:**

To determine the prevalence of suicidal behaviour and the risk factors associated with suicidal behaviour, among patients with CCF in Nigeria.

**Setting:**

Cardiology outpatient clinic of Lagos State University Teaching Hospital, Lagos, Nigeria.

**Methods:**

A cross-sectional study was conducted among 98 randomly selected patients with a diagnosis of CCF. Participants were assessed with a socio-demographic and clinical factors questionnaire and Beck Scale of Suicidal Ideation. Chi-square test, *t*-test and logistic regression were used to analyse data.

**Results:**

The prevalence of suicidal ideation and suicidal attempt among patients with CCF was 52% and 1%, respectively. No socio-demographic factor was significantly associated with suicidal ideation. Clinical factors associated with suicidal ideation were age at diagnosis (*p* = 0.042), aetiology of CCF (*p* = 0.001) and severity of CCF (*p* = 0.032). Only the severity of CCF (odds ratio [OR] = 20.557, *p* = 0.014) predicted suicidal ideation among patients with CCF.

**Conclusion:**

Suicidal behaviour constitutes a huge burden among the outpatient CCF population. The identification of clinical risk factors for suicidal ideation (age at diagnosis, aetiology and severity of CCF) further illuminates a pathway to mortality among patients with CCF.

**Contribution:**

The findings lend a voice to the need for screening for suicidal behaviour, suicide prevention programmes, surveillance systems and government policies that support mental health for patients with CCF.

## Introduction

Congestive cardiac failure (CCF) is a chronic, progressive, debilitating and non-communicable disease.^1,2^ It is described by the American Heart Association (AHA) as a complex clinical syndrome that develops as a result of impaired filling or ejection of blood from the ventricles – be it a functional or structural impairment.^1^ It is usually the outcome of many cardiac diseases^1,3^ with its principal symptoms being difficulty in breathing, exhaustion and fluid retention.^1,4,5^ Congestive cardiac failure is estimated to globally affect more than 26 million people.^6^ This number is expected to increase as more people survive cardiac diseases and live longer.^6^ While regional variations in the burden of CCF have been established, there is a paucity of information on its prevalence in the African population including Nigeria.^3,7^ Mortality from CCF is high and is worse in Africa where 34% of global CCF deaths occur.^8^ In sub-Sahara Africa (SSA), the estimated 6-month death rate from CCF is 18%.^3,9^ A significant number of deaths of patients with CCF result from suicide^10,11^ and penultimate to these suicide deaths is an increased propensity to other suicidal behaviour.^9^

Suicidal behaviour is a complex phenomenon that embodies suicidal thoughts or ideation, planning suicide, suicide attempt and completed suicide.^12,13^ Suicidal ideation is recurrent thoughts of ending one’s life and suicidal plans involve mapping out a method of ending one’s life. In a suicidal attempt, potentially self-injurious activities are carried out with at least some intention to die and ultimately complete suicide is the fatal outcome of the self-injurious activities.^12^ These behaviours are of public health importance,^14,15^ and increasing evidence has linked CCF with a higher risk of suicidal behaviour.^9,16,17,18^ A cross-sectional study among CCF patients reported suicidal ideation in 17.1% of the sample.^17^ This is much higher compared to the global lifetime prevalence of suicidal behaviour found to be 9.2%.^19^

Despite this burden, the prevalence of suicidal behaviour among persons with CCF in developing nations such as Nigeria is not known. In addition, few studies have examined the risk factors for suicidal behaviour among patients with CCF. The identified risk factors are mainly psychological factors.^9,17,18^ Based on a diligent search of electronic literature, there is a dearth of studies that have examined possible socio-demographic and clinical correlates for suicidal behaviour among patients with CCF. A study identified the age of CCF patients as a risk factor for suicidal behaviour; however, on multivariate analysis, age was not predictive of suicidal behaviour.^17^ The study also did not examine other socio-demographic risk factors.^17^ In the same vein, the study reported no significant association between the aetiology of CCF, the New York Heart Association (NYHA) functional class of CCF and the presence of suicidal ideation.^17^ The few studies that explored the relationship of these factors to suicidal behaviour among persons with CCF have been in the Western world, and the findings are more likely to be cross-culturally different from developing nations.^11,17^

Addressing these research gaps should result in a more representative data for suicidal behaviour among patients with CCF in Nigeria and would highlight the magnitude and extent of the phenomena among this population. This data will be essential for health resources planning for health policymakers. It will also assist in the prioritisation of services for this population. Further, the identification of socio-demographic as well as clinical factors that are associated with suicidal behaviour among this population will provide a basis for interventions with considerable impact on the quality of life, work productivity and reduction of the burden of caregivers.

This study therefore aimed to determine the socio-demographic and clinical risk factors associated with suicidal behaviour among patients with CCF in Nigeria.

## Research methods and design

### Study design

The study adopted a cross-sectional design.

### Setting

The study was carried out at the cardiology outpatient clinic of Lagos State University Teaching Hospital, Lagos, Nigeria. An average of 150 patients with CCF regularly attended the clinic yearly.

### Study population and sampling strategy

The participants were adults with a diagnosis of CCF. The inclusion criteria included a diagnosis of CCF made by a consultant cardiologist and confirmed by an echocardiogram scan as provided in the patient’s hospital record and consent to participate in the study. Those with a prior history of mental illness before CCF diagnosis (psychosis, mood disorder and anxiety) and illness that is severe enough to prevent neuropsychiatry assessment (e.g. patient on cardiopulmonary support) were excluded from the study.

The sample size was calculated as 98 from a finite population of 150 regular follow-up patients, using a prevalence of 17.1% (prevalence of suicidal ideation among CCF patients)^17^ at a 95% confidence interval in detecting a margin of error of 0.05.

Participants were selected using a simple random sampling technique (ballot method).^20^ On each clinic day, the total number of patients who met the eligibility criteria comprised the sampling frame for the day and it equalled the total number of ballot papers put in the ballot box for the day. The number of ‘YES’ ballot papers was the number of participants to be recruited for the day (10), while the number of ‘NO’ ballot papers was determined by subtracting the number of ‘YES’ ballot papers (10) from the sampling frame. Each patient was allowed to pick one ballot paper from the ballot box, giving each person an equal chance to participate in the study or otherwise. Only patients who picked a ‘YES’ were recruited into the study.

### Data collection

Data collection was done using the following instruments:

- A Pro-forma questionnaire was used to collect relevant socio-demographic and clinical information. Socio-demographic factors included in the questionnaire were age, gender, religion, marital status, the highest level of education, employment status and type of occupation. Clinical factors included were the aetiology of CCF, precipitants of CCF, severity of CCF, duration of illness and presence and number of chronic medical comorbidities.
- Beck Scale for Suicidal Ideation (BSSI) was used to assess suicidal behaviour.

Beck Scale for Suicidal Ideation is one of the most commonly used instruments for evaluating suicidal behaviour.^21^ It is self-administered and takes 10 min to be completed,^22,23^ and it assesses both the presence and severity of suicidal behaviour in the past week.^24^ It is a 21-item questionnaire, the first five questions screen for suicidal behaviour and only participants who have a score of at least one on questions four or five – indicating passive or active suicide ideation will complete the next 14 questions.^22,25,26^ These initial 19 questions are rated on a scale from 0 to 2 yielding a maximum score of 38. Generally, the higher the total score, the higher the severity of suicidal ideation.^26^ In this study, only 51 participants had suicidal ideation based on the screening subsection of BSSI; hence they were the ones that completed the next 14 questions. This proportion that completed the questionnaire indicated the prevalence of suicidal ideation in the study. The last two items assess the number of previous suicide attempts and the seriousness of the intent to die associated with the last attempt.^25^ All participants are to answer the last two questions.

The BSSI have acceptable reliability and validity.^24,27,28^ It has also been used in Nigerian studies.^25,29^

The diagnosis of CCF was made by consultant cardiologists, while echocardiogram scan was reported by consultant interventional cardiologists. These information were available in the case files of patients with CCF. The pro-forma questionnaire was administered to each participant. Only those who were employed (49 participants) were further asked about their type of occupation. The participants were offered necessary guidance to complete the BSSI. The NYHA class, which is a measure of CCF severity, was obtained from the case file of the participants. Participants with suicidal behaviour were psycho-educated by the researchers and they were also brought to the notice of the attending doctor for appropriate referral.

### Data analysis

The data were coded, entered into the Statistical Package for Sociological Sciences (SPSS version 26), cleaned and analysed.

The independent variables were the socio-demographic factors (age, gender, religion, marital status, the highest level of education, employment status and type of occupation) and clinical factors (aetiology of CCF, precipitants of CCF, class or severity of CCF, duration of illness and comorbidities).

The dependent variables were suicidal ideation and suicide attempt.

Categorical variables were summarised with frequencies and percentages while continuous variables were summarised with their mean and standard deviation.

Bivariate analysis was conducted to compare socio-demographic and clinical factors between CCF patients with suicidal behaviour and those without suicidal behaviour using appropriate statistics such as the chi-square (χ^2^) test of independence for categorical (with Fisher exact corrections where appropriate). For normally distributed quantitative variables, an independent *t*-test was used while the Mann–Whitney U test was used to analyse the quantitative variables that were not normally distributed.

Multivariate logistic regression analysis was used to determine which of the significant socio-demographic, clinical and psychological factors in bivariate analyses were independently predictive of suicidal behaviour among persons with CCF. The level of significance (*p*-value) was set at 0.05.

### Informed consent

Written informed consent was obtained from all individual participants involved in the study. This was after the details and potential benefits of the proposed study were explained to them. The anonymity, confidentiality and optional participation and withdrawal from the study were also explained.

### Ethical considerations

An application for full ethical approval was made to the Ethics and Research Committee of a large South-western tertiary hospital in Nigeria and ethical consent was received on 28 February 2022. The ethical approval number is (LREC/06/10/1775). All procedures performed in this study involving human participants were in accordance with the ethical standards of the institutional and national research committee and with the 1964 Helsinki Declaration and its later amendments or comparable ethical standards.

## Results

A total number of 98 questionnaires administered to the participants were retrieved and analysed, representing a 100% response rate.

### Socio-demographic characteristics of the respondents

As shown in Table 1, the mean age of the respondents was 54.05 ± 17.13 years. More (56.1%) of the respondents were females and a majority (78.6%) were married. Proportionately more of the respondents were Christians and attained tertiary education level. Half of the respondents were employed, more of whom were sales and service workers.

**TABLE 1:** Socio-demographic variables of respondents (*N* = 98).

Socio-demographic variables	Frequency	%	Mean ± s.d.
**Age (years)**			54.05 ± 17.13
Young (18–39)	24	24.5	-
Middle (40–59)	31	31.6	-
Elderly (≥ 60)	43	43.9	-
**Gender**			
Male	43	43.9	-
Female	55	56.1	-
**Religion**			
Christianity	61	62.2	-
Islam	37	37.8	-
**Marital status**			
Single	9	9.2	-
Married	77	78.6	-
Separated or divorced	3	3.0	-
Widowed	9	9.2	-
**Level of education**			
No or primary education	19	19.4	-
Secondary	34	34.7	-
Tertiary	45	45.9	-
**Employment status**			
Unemployed	30	30.6	-
Employed	49	50.0	-
Retired	19	19.4	-
**Occupation (*n* = 49)**			
Managers	2	2.0	-
Professionals	14	14.3	-
Technicians and associate professionals	5	5.1	-
Clerical support workers	3	3.1	-
Service and sales workers	19	19.4	-
Skilled agricultural, forestry and fishery workers	1	1.0	-
Craft and related trades workers	2	2.0	-
Elementary occupations	3	3.1	-

s.d., standard deviation.

### Clinical characteristics of the respondents

As shown in Table 2, majority of participants were diagnosed with CCF in their middle or old age and a larger proportion, (39.8%) were diagnosed within the last 12 months. More than half of the participants had CCF attributed to hypertensive heart disease while chest infection precipitated CCF in 29.6% of the respondents. Many of the study participants had NYHA functional class II of CCF. Fifty-three (54.1%) of the participants had other chronic medical comorbidities of which diabetes mellitus was the most common. Proportionately majority, 42 (79.2%) had single comorbidity. Of the 53 participants who had chronic medical comorbidities, 11 reported two comorbidities each; hence number of chronic medical comorbidities was 64.

**TABLE 2:** Clinical history variables of respondents (*N* = 98).

Clinical history variables	Frequency	%	Mean ± s.d.
**Age at diagnosis (years)**			51.55 ± 16.36
Young (0–39)	26	26.5	-
Middle-Age (40–59)	35	35.7	-
Elderly (≥ 60)	37	37.8	-
**Duration of illness (months)**			
0–12	39	39.8	-
13–36	33	33.7	-
≥ 37	26	26.5	-
**Aetiology of CCF**			
Hypertensive heart disease	52	53.1	-
Ischaemic heart disease	8	8.2	-
Dilated cardiomyopathy	16	16.3	-
Rheumatic heart disease	12	12.2	-
Congenital heart disease	8	8.2	-
Peripartum cardiomyopathy	2	2.0	-
**Precipitant of CCF**			
Atrial fibrillation	24	24.5	-
Chest infection	29	29.6	-
Uncontrolled hypertension	14	14.2	-
Disease progression	5	5.1	-
Drug non-compliance	18	18.4	-
Pulmonary embolism	7	7.1	-
Thyrotoxicosis	1	1.0	-
**Functional class of CCF NYHA**			
I	13	13.3	-
II	57	58.1	-
III	14	14.3	-
IV	14	14.3	-
**Presence of medical comorbidities**			
Yes	53	54.1	-
No	45	45.9	-
**Medical comorbidities (*n* = 64)**			
Diabetes mellitus	31	58.5	-
Asthma	23	43.4	-
COPD	7	13.2	-
Others	3	5.7	-
**No of medical comorbidities (*n* = 53)**			
Single	42	79.2	-
Multiple	11	20.8	-

s.d., standard deviation; CCF, congestive cardiac failure; NYHA, New York Heart Association; COPD, chronic obstructive pulmonary disease.

### Prevalence of suicidal ideation and suicidal attempt

As assessed with BSSI, the proportion of respondents who had suicidal ideation was 52.0% (51 respondents) while the proportion of respondents who had at least one suicide attempt was 1% (one respondent).

### Socio-demographic factors associated with suicidal ideation among participants

Using chi-square test, there was no statistically significant association between suicidal ideation among the patients and age, gender, religion, marital status, the highest level of education, employment status and type of occupation as all the *p*-values of these variables exceeded the 0.05 level of significance as shown in Table 3. This implies that these socio-demographic variables were not significantly associated with suicidal ideation among the participants.

**TABLE 3:** Socio-demographic factors associated with suicidal ideation among participants.

Socio-demographic variables	Suicidal ideation				*df*	χ^2^	*p*
	Yes (*N* = 51)		No (*N* = 47)				
	*n*	%	*n*	%			
**Age (years)**	-	-	-	-	2	3.940	0.139
Young (18–39)	16	31.4	8	17.0	-	-	-
Middle (40–59)	17	33.3	14	29.8	-	-	-
Elderly (≥ 60)	18	35.3	25	53.2	-	-	-
**Gender**	-	-	-	-	1	1.375*	0.241
Male	19	37.3	24	51.1	-	-	-
Female	32	62.7	23	48.9	-	-	-
**Religion**	-	-	-	-	1	0.885	0.347
Christianity	34	66.7	27	57.4	-	-	-
Islam	17	33.3	20	42.6	-	-	-
**Marital status**	-	-	-	-	4	6.932†	0.075
Single	8	15.7	1	2.1	-	-	-
Married	36	70.6	41	87.2	-	-	-
Separated	1	2.0	1	2.1	-	-	-
Divorced	1	2.0	0	0.0	-	-	-
Widowed	5	9.8	4	8.5	-	-	-
**Level of education**	-	-	-	-	3	0.361†	0.987
No or primary education	10	19.6	9	19.2	-	-	-
Secondary	17	33.3	17	36.2	-	-	-
Tertiary	24	47.1	21	44.7	-	-	-
**Employment status**	-	-	-	-	2	4.514	0.105
Unemployed	20	39.2	10	21.3	-	-	-
Employed	24	47.1	25	53.2	-	-	-
Retired	7	13.7	12	25.5	-	-	-
**Occupation (*n* = 49)**	-	-	-	-	7	6.756	0.450†
Managers	0	0.0	2	8.0	-	-	-
Professionals	6	25.0	8	32.0	-	-	-
Technicians and associate professionals	3	12.5	2	8.0	-	-	-
Clerical support workers	2	8.3	1	4.0	-	-	-
Service and sales workers	8	33.3	11	44.0	-	-	-
Skilled agricultural, forestry and fishery workers	1	4.3	0	0.0	-	-	-
Craft and related trades workers	2	8.3	0	0.0	-	-	-
Elementary occupations	2	8.3	1	4.0	-	-	-

Note: *p*-values are significant at 0.05 significance level.

† , *F*–statistic and p-values from Fisher’s exact test;

* , Y–statistic and *p*-values from Yate’s continuity correction.

### Clinical factors associated with suicidal ideation among participants

Using the chi-square test, age at diagnosis (χ^2^ = 6.319, *df* = 2, *p* = 0.042), aetiology of CCF (χ^2^ = 17.756, *df* = 5, *p* = 0.001) and functional class of CCF (χ^2^ = 8.778, *df* = 3, *p* < 0.032) were found to be significantly associated with suicidal ideation among the participants as shown in Table 4. In contrast, there was no statistically significant association between suicidal ideation among the participants and duration of illness, precipitants of CCF, presence of comorbidity and the number of comorbidities in this study.

**TABLE 4:** Clinical factors associated with suicidal ideation among participants.

Clinical variables	Suicidal ideation				*df*	χ^2^	*p*
	Yes (*N* = 51)		No (*N* = 47)				
	*n*	%	*n*	%			
**Age at diagnosis (years)**	-	-	-	-	2	**6.319**	0.042*
Young (0–39 years)	19	37.3	7	14.9	-	-	-
Middle (40–59 years)	16	31.4	19	40.4	-	-	-
Elderly (> 60 years)	16	31.4	21	44.7	-	-	-
**Duration of illness (months)**	-	-	-	-	2	1.368	0.505
0–12 months	23	45.1	16	34.0	-	-	-
13–36 months	15	29.4	18	38.3	-	-	-
≥ 37 months	13	25.5	13	27.7	-	-	-
**Aetiology of CCF**	-	-	-	-	5	**17.765†**	0.001*
Hypertensive heart disease	18	35.3	34	72.3	-	-	-
Ischaemic heart disease	6	11.8	2	4.3	-	-	-
Dilated cardiomyopathy	13	25.5	3	6.4	-	-	-
Rheumatic heart disease	8	15.7	4	8.5	-	-	-
Congenital heart disease	6	11.8	2	4.3	-	-	-
Peripartum cardiomyopathy	0	0.0	2	4.3	-	-	-
**Precipitant of CCF**	-	-	-	-	7	5.760†	0.589
Atrial fibrillation	11	21.6	13	27.7	-	-	-
Chest infection	18	35.3	11	23.4	-	-	-
Uncontrolled hypertension	5	9.8	2	4.3	-	-	-
Disease progression	2	3.9	3	6.4	-	-	-
Drug non-compliance	8	15.7	10	21.3	-	-	-
Pulmonary embolism	4	7.8	3	6.4	-	-	-
Thyrotoxicosis	1	2.0	0	0.0	-	-	-
Dietary indiscretion	2	3.9	5	10.6	-	-	-
**Functional class of CCF NYHA**	-	-	-	-	3	**8.778**	0.032*
I	7	13.7	6	12.8	-	-	-
II	24	47.1	33	70.2	-	-	-
III	8	15.7	6	12.8	-	-	-
IV	12	23.5	2	4.3	-	-	-
**Presence of medical comorbidities**	-	-	-	-	1	0.193‡	0.661
Yes	26	51.0	27	57.4	-	-	-
No	25	49.0	20	42.6	-	-	-
**No of medical comorbidities (*n* = 48)**	-	-	-	-	1	0.043‡	0.836
Single	19	82.6	19	76.0	-	-	-
Multiple	4	17.4	6	24.0	-	-	-

CCF, congestive cardiac failure; NYHA, New York Heart Association.

* , *p* = level of significance < 0.05 (bold values indicate statistical significance).

† , *F*-statistic and *p*-values from Fisher’s exact test;

‡ , Y-statistic and *p*-values from Yate’s continuity correction.

### Factors independently associated with suicidal ideation among patients with congestive cardiac failure

The evaluated logistic regression model presented in Table 5 reveals that of the three clinical-related variables shown to be significantly associated with suicidal ideation from the chi-square analysis, only the functional class of CCF was predictive of suicidal ideation.

**TABLE 5:** Logistic regression of clinical correlates for suicidal ideation among participants.

Variables	B	Odds ratio	95% CI	*p*
**Constant**	−18.953	5.87E-09	-	0.999
**Age at diagnosis (years)**				
Young (0–39 years)	0.349	1.417	0.208–9.664	0.722
Middle (40–59 years)	−0.802	0.449	0.116–1.735	0.245
Elderly (reference)	-	-	-	-
**Aetiology of CCF**				
Hypertensive heart disease	21.537	2.26E+10	0.000–0.000	0.999
Ischaemic heart disease	23.218	1.21E+11	0.000–0.000	0.999
Dilated cardiomyopathy	24.112	2.96E+11	0.000–0.000	0.999
Rheumatic heart disease	22.719	7.36E+09	0.000–0.000	0.999
Congenital heart disease	23.262	1.27E+10	0.000–0.000	0.999
Peripartum cardiomyopathy (reference)	-	-	-	-
**Functional class of CCF**				
I (reference)	-	-	-	-
II	0.925	2.523	0.484–13.138	0.272
III	1.372	3.944	0.443–35.107	0.219
IV	3.024	20.577	1.863–227.259	**0.014**

CI, confidence interval; CCF, congestive cardiac failure.

Note: Bold *p*-values are significant at 0.05 level of significance; omitted categories are reference categories, Nagelkerke *r*^2^ = 0.545.

The estimation showed that compared to the functional class I of CCF, being functional, class IV of CCF makes it 20.577 times more likely for a patient with CCF to have suicidal ideation, with OR = 20.557 (95% CI, 1.863 – 227.259, *p* = 0.014).

The Nagelkerke *r*-square indicates that the estimated model was able to estimate 54.5% of the variations in the probability of patients with CCF having suicidal ideation. The percentage of correct prediction of patients with CCF having suicidal ideation by the model (featuring the clinical-related and psychological-related variables) is 80.6%.

## Discussion

This study found that the prevalence of suicidal ideation and suicidal attempt among patients with CCF was 52% and 1%, respectively. No socio-demographic factor was significantly associated with suicidal ideation; however, clinical factors associated with suicidal ideation were age at diagnosis, aetiology of CCF and severity of CCF.

The prevalence of suicidal ideation among patients with CCF in this study was 52%. This is three times the prevalence of 17.1% reported in a study done in Germany, which explored suicidal ideation among patients with CCF.^17^ An American cross-sectional study examined suicidal ideation among adults with cardiovascular disease and found that patients with CCF had the highest suicidal ideation rate of 10.9%.^18^ The marked difference in the prevalence of suicidal ideation in this study and those of previous studies may be because of the different instruments used to screen for suicidal ideation. Previous studies assessed suicidal ideation using PHQ-9, which evaluates passive thoughts of self-injury or death within the past 2 weeks.^17,18,30^ In addition, as the previous studies were done in developed countries with better health care services, there may also be cross-cultural disparities in the psychosocial impact as well as the emotional response of patients in developing countries to a chronic illness such as CCF.^31^

This study found the prevalence of suicidal attempts to be 1% among the outpatient CCF population. To the knowledge of the author, no study has estimated the prevalence of suicidal attempts among patients with CCF. However, other cardiovascular diseases including atrial fibrillation, angina and myocardial infarction have been associated with a higher risk of suicidal attempts.^32,33^ The prevalence of suicidal attempts in this study is lower than that which was reported in a global study among healthy adults, which is 2.7%.^14^ It is, however, slightly higher than the pooled lifetime prevalence of 0.7% recorded in a systematic review of suicidal behaviour across the Lifespan in Nigeria.^19,34^ This may point to the effect of physical illness in this study’s population. Given the relatively low prevalence of suicidal attempts when compared to the suicidal ideation prevalence found in this study, it is reasonable to deduce that some protective factors may be at play. In Nigeria, it has been found that religious and cultural dispositions may prevent individuals from attempting suicide despite having thoughts of suicide.^35,36^ However, because of the stigma associated with suicidal behaviour in this cultural setting, under-reporting of suicidal attempts may be the case in this population.

None of the socio-demographic-related factors of patients with CCF considered in this study was found to be significantly associated with suicidal ideation. This agrees with a related study that found no association between gender and suicidal ideation.^17^ However, the same study found a significant association between age and suicidal ideation in bivariate but not in regression analysis.^17^ In comparison, a community survey of healthy adults in Nigeria reported an independent association between suicidal ideation and female gender, older age, low occupational group and those not married.^37^ The dynamic effect of the presence of a chronic medical illness – CCF in this case – in the development of suicidal behaviour can readily account for this contrasting finding.

Age at diagnosis was found to be a significant correlate of suicidal ideation among the patients. However, with multivariate analysis, it was not predictive of suicidal ideation. No previous study was found that examined the relationship between age at diagnosis of CCF and suicidal ideation. More than two-thirds of participants in this study were diagnosed with CCF at a young and middle age corresponding to the productive years of the affected persons. Functional limitations imposed by CCF at this stage of life could explain the relationship between age at diagnosis and the development of suicidal ideation as well as its severity. Further, the youngest age group in this study were also those with the lowest age at diagnosis. The aetiology of CCF in this age group was mainly congenital heart disease indicating the lifelong duration of illness. The chronicity of ill-health may inform the severity of suicidal ideation.

Aetiology of CCF was significantly associated with, but not predictive of suicidal ideation among the patients in this study. A related study reported no significant association between suicidal ideation and the aetiology of CCF, which is in contrast to the finding in this study.^17^ The regional difference in the prevalent aetiology of CCF may readily explain the contrast in the findings in this study and the previous study.

New York Heart Association functional class of CCF was also found to be a significant correlate of suicidal ideation among the patients. After multivariate analysis, it was also found to be predictive of suicidal ideation among them. Compared to the functional class I of CCF, being functional class IV of CCF makes it 20 times more likely for a patient with CCF to have suicidal ideation. This finding is in contrast to the study done in Germany, which found no significant association between suicidal ideation and NYHA functional class.^17^ Cross-cultural differences in the prevalent NYHA functional class may explain the contrast in the findings in this study and the previous study. Furthermore, patients with NYHA functional class IV of CCF are unable to carry out any physical activity without symptoms of CCF, and they also experience symptoms of CCF at rest.^1^ This marked distress and limitation of physical activity experienced at this stage is a ready substrate for thoughts of suicide.^38^

Suicidal ideation is a reversible precursor of suicide, especially when it is identified early and its antecedents attended to.^39^ The finding in this study thus points out the need to routinely screen all patients with signs and symptoms of CCF not only at presentation but at different stages of their follow-up in the clinic. Although many patients who verbalise suicidal ideation may never kill themselves, communications of thoughts of suicide should not be ignored by the attending clinician as it indicates psychological distress.^39^ Hence, the need for in-depth evaluation of such a patient’s mental state cannot be over-emphasised.

Given the preponderance of suicidal ideation observed among patients with CCF in this study, the non-association of any socio-demographic factor with suicidal ideation in this patient group further underscores the need for screening of all patients with CCF for suicidal behaviour, irrespective of their age, gender, marital status, education level or employment status. However, this should not undermine the required high index of suspicion and particular attention to be paid to those with the general population-established socio-demographic correlates of suicidal behaviour.

The identification of age at onset of illness, aetiology and NYHA functional class of CCF as vulnerability factors in the development of suicidal ideation has clinical implications. Firstly, it calls for detailed history-taking while assessing patients with CCF. Rather than just asking for the duration of symptoms, deducing a young age at the onset of the illness from history may prompt the clinician to expect possible psychological disturbance in the patient. Secondly, the underlying causes of CCF often persist as a comorbidity with CCF, thus adding to the burden of the illness. In addition, the marked limitation of functioning posed by increasing severity of CCF as depicted by NYHA classification may be expected to impact on quality of life of the patients. The above underscores the need for psychological evaluation of patients with CCF.

The paucity of studies on socio-demographic correlates of suicidal behaviour among patients with CCF highlights the need for more research into this relationship. There is also a possibility of different findings if this study was done among patients with CCF who are on admission and/or with a larger sample size. These are research gaps that are worthy of exploration.

Being a hospital-based study of outpatient clinic attendees may limit the generalisability of the findings to the in-patient and the general population of individuals with CCF. The cross-sectional design of the study also limits possible inferences on causality. The strength of this study, however, lies in its use of a random sampling technique, which reduced the chance of selection bias. The study adopted the use of standardised instruments to assess suicidal behaviour among the participants. Further, the findings of this study could serve as a baseline for comparison in future studies.

## Conclusion

Based on the analysis of data obtained, it can be concluded that suicidal ideation constitutes a huge burden among the outpatient CCF population. While no socio-demographic risk factor was identified, clinical factors (age at diagnosis, aetiology of CCF and NYHA functional class of CCF) are important risk factors associated with suicidal ideation in the study population.

The identification of the risk factors for suicidal ideation further illuminates a pathway to mortality among patients with CCF. The findings lend a voice to the need for screening for suicidal behaviour among patients with chronic medical illnesses, consultation liaison with mental health physicians, suicide prevention programmes, surveillance systems and government policies that support mental health. Further, this study addresses the information deficit on the prevalence and risk factors for suicidal behaviour among patients with CCF.
